# Risk factors for thrombotic events in systemic lupus erythematosus patients with antiphospholipid antibodies: insights from morphometric measurements of carotid arteries

**DOI:** 10.3389/fcvm.2024.1373097

**Published:** 2024-06-26

**Authors:** Qing Yang, Qi Liu, Changqing Yin, Xiaoyu Zhang, Xi Chen, Dmytro Pylypenko, Hao Chen, Qiang Shu, Dexin Yu

**Affiliations:** ^1^ Department of Radiology, Qilu Hospital, Cheeloo College of Medicine, Shandong University, Jinan, China; ^2^ Department of Rheumatology, Qilu Hospital, Cheeloo College of Medicine, Shandong University, Jinan, China; ^3^ Department of Rheumatology, Qilu Hospital, Shandong Provincial Clinical Research Center for Immune Diseases and Gout, Jinan, China; ^4^ Department of Orthopaedics, Peking University Third Hospital, Beijing, China; ^5^ GE Healthcare, MR Research China, Beijing, China; ^6^ Clinical Epidemiology Unit, Clinical Research Center of Shandong University, Qilu Hospital, Cheeloo College of Medicine, Shandong University, Jinan, China

**Keywords:** carotid artery, high-resolution magnetic resonance imaging, systemic lupus erythematosus, antiphospholipid antibodies, thrombosis, morphometry

## Abstract

**Objective:**

To identify the correlation between thrombosis and atherosclerosis in systemic lupus erythematosus (SLE) patients with antiphospholipid antibodies (aPLs) (SLE/aPLs) through high-resolution magnetic resonance imaging (HR-MRI) of the carotid artery.

**Methods:**

A single-center, cross-sectional study was conducted. We collected consecutive patients with SLE/aPLs and healthy controls who underwent carotid HR-MRI examinations. The morphometric characteristics of the common carotid artery (CCA), internal carotid artery (ICA), external carotid artery (ECA), and carotid bulb (Sinus) were measured, and the differences in morphometric parameters between different groups were analyzed.

**Results:**

A total of 144 carotid arteries were analyzed. Compared with the control group, the wall area, wall thickness (WT and WTmax), and normalized wall index of CCA, ICA, ECA, and Sinus were increased in patients with SLE/aPLs, and the total vascular area (TVA) of CCA, ICA, and Sinus, and the bifurcation angle (BIFA) of ICA-ECA were also increased. A negative lupus anticoagulant (LAC) (with or without positive anticardiolipin antibody (aCL) or anti-β2glycoprotein antibody (aβ2GPI)) contributed to illustrating lower increased TVA and thickened vessel walls of CCA and ICA in SLE/aPLs patients without thrombotic events. Logistic regression analysis showed that WTmax_Sinus_ and WTmax_Global_ were independent risk factors for thrombotic events in SLE/aPLs patients. The receiver operator characteristic curve showed that the cut-off value of WTmax_Sinus_ was 2.855 mm, and WTmax_Global_ was 3.370 mm.

**Conclusion:**

HR-MRI ensures the complete and accurate measurement of carotid morphometric parameters. Compared with the control group, the carotid artery in patients with SLE/aPLs is mainly characterized by diffusely thickened vessel walls, and the patients with thrombotic events showed additional higher vascular area of CCA and ICA, and BIFA of ICA-ECA without significant change in lumen area. The carotid arteries of SLE/aPLs patients with thrombotic events exhibited significant vessel wall thickening in all segments except ECA compared to those without thrombotic events. LAC-negative and non-thrombotic events distinguish relatively early atherosclerosis in the carotid arteries in patients with SLE/aPLs. Patients with SLE/aPLs that possess circumscribed thickened carotid vessel walls (>3.370 mm), particularly thickened at the Sinus (>2.855 mm), may require management strategies for the risk of thrombotic events.

## Introduction

Antiphospholipid syndrome (APS) is a rare acquired autoimmune disease characterized by recurrent thrombosis and/or pregnancy complications, with the persistent existence of one or more antiphospholipid antibodies (aPLs), including lupus anticoagulant (LAC), anticardiolipin antibody (aCL), and anti-β2glycoprotein antibody (aβ2GPI) (1, 2). Acquired thrombosis is one of the hallmarks of the onset and progression of APS symptoms (3). Secondary APS is often associated with autoimmune rheumatic diseases, particularly in individuals with systemic lupus erythematosus (SLE) (4, 5). SLE patients with positive aPLs (SLE/aPLs) are associated with a more severe disease course, acquired organ damage, and higher cardiovascular disease risk (6–8). However, currently, there is a lack of relevant research on the susceptibility characteristics of thrombosis in patients with SLE/aPLs.

Recent studies suggest that the pathophysiology of thrombosis may accelerate atherosclerosis in patients with APS (9–11) and that traditional risk factors for atherosclerosis are closely associated with the prevalence and mortality of APS and thrombosis (6, 12, 13). Some studies have observed increased intima-media thickness (IMT) of the carotid artery and atherosclerotic plaques in patients with APS or aPLs carriers (12–15). In contrast, others have not observed a difference between IMT of the carotid artery in patients with APS and healthy individuals (16–18). It is well-recognized that the method of measuring IMT using Doppler ultrasound is relatively primary and limited, which may be the reason for the contradictory conclusions obtained by different studies. Therefore, it is essential to comprehensively and systematically evaluate the morphometric characteristics of critical regions of the carotid arteries in patients with SLE/aPLs.

High-resolution magnetic resonance imaging (HR-MRI) is an emerging auxiliary examination technique that can clearly identify carotid and cerebral arterial lesions that are difficult to discern using standard imaging techniques. It can better characterize the structure of the vessel wall and, combined with image reconstruction, provide accurate morphometric measurements of the entire scanned region of interest for researchers (19–21). Based on this, we believe that HR-MRI can contribute to a comprehensive and systematic morphometric evaluation of the carotid arteries in patients with SLE/aPLs, aiming to overcome the limitations of measurement parameters and sites in carotid Doppler ultrasound. However, there are currently no relevant applications or reports available.

The purpose of this study is to investigate the characteristic morphometric changes of the carotid arteries by measuring the wall and lumen structure of the internal carotid artery (ICA), external carotid artery (ECA), common carotid artery (CCA), and carotid bulb (Sinus) in patients with SLE/aPLs using HR-MRI, thereby elucidating the intrinsic connection between the morphometric characteristics of the carotid arteries and thrombosis from the perspective of radiology and providing a theoretical basis and data to support the early screening and clinical prevention and treatment of thrombosis in patients with SLE/aPLs.

## Materials and methods

### Study population

This study was approved by the Institutional Review Committee of Qilu Hospital of Shandong University (#KYLL-202208-048), and the study protocol conformed to the ethical guidelines of the 1975 Declaration of Helsinki as reflected in *a priori* approval by the institution's human research committee. All participants fully satisfied the patient's right to know and signed the informed consent form before enrolling in the study.

Inpatients who underwent a carotid HR-MRI examination from June 2021 to October 2023 at Qilu Hospital of Shandong University were prospectively and continuously selected to participate in this cross-sectional study. Patients enrolled in the SLE/aPLs group were over 18 years old and fulfilled the SLE/aPLs criteria, which referred to meeting the 2019 EULAR/ACR SLE classification criteria (22) and having positive aPLs. The patients' clinical information was recorded in detail to determine whether it met the revised Sapporo classification criteria for APS (1). The “clinical non-criteria manifestation” described by Gilberto et al. in 2020 (23) was also recorded, considering that our understanding of APS is constantly improving. Patients in the SLE/aPLs group were further classified into the SLE/aPLs with thrombosis group (thrombosis group) and the SLE/aPLs without thrombosis group (non-thrombosis group) based on whether they had objectively documented or ongoing symptomatic or asymptomatic thrombotic events. Patients suffering malignancy or hematologic disorders, or having a history of non-atherosclerotic brain and carotid artery diseases such as Moya-Moya disease, dissection, and fibromuscular dysplasia, or previous carotid intervention such as arterial stenting, end-arterial decortication surgery, and neck radiation therapy, or with any contraindications to MR examination were excluded from this study. The control group included healthy participants during the same period without any history of autoimmune disease or thrombosis. These participants received extra HR-MRI scans after satisfying the right to know fully and obtaining consent from the patients and their accompanying relatives.

### Data collection

Demographic, clinical, and laboratory data were collected and recorded, and the interval between the record and the HR-MRI scan was less than two weeks. In addition to documented thrombotic events and course of disease, traditional cardiovascular risk factors such as arterial hypertension, hyperlipidemia, diabetes mellitus, obesity (body mass index (BMI) ≥ 30 kg/m^2^), smoking habits, and previous and/or current treatment were collected.

Criteria for positive aPLs: aPLs must be positive in two or more tests with intervals of more than 12 weeks. Anticardiolipin antibodies (IgG/IgM) were determined by enzyme-linked immunosorbent assay (ELISA) and were considered positive with the presence of medium/high titer (>40/>80 IgG phospholipid (GPL) unites or >40/>80 IgM phospholipid (MPL) unites) (13, 24–26). Anti-β2glycoprotein antibodies (IgG/IgM) were detected by chemiluminescence assay (CIA) and were considered positive with >7.4 chemiluminescence units (CU) for IgG or >3.6 CU for IgM. LAC was identified using the diluted Russell Viper Venom Time and was considered positive with a normalized ratio (NR) ≥1.2. The number of positive aPLs was classified as single-positive aPLs, double-positive aPLs, and triple-positive aPLs (13, 24–29). The Department of Clinical Laboratory of Qilu Hospital of Shandong University tested and reported all the samples and set the reference range of the positive criteria according to the internal quality control.

### Imaging protocol

All MRI scans were performed using a Discovery 750 3.0T magnetic resonance scanner (General Electric Medical System, WI, USA), equipped with a dedicated 40-channel cerebral vascular and carotid integrated coil (Medcoil Healthcare Co Ltd, Suzhou, China).

A three-dimensional (3D) T1- and T2-weighted HR-MRI system was used to analyze the geometry and vessel walls of carotid arteries. The scanning range of the 3D-CUBE sequence completely covered the arterial vascular structure at the bifurcation of the carotid arteries on both sides. The 3D-CUBE-T1 weighted imaging parameters were as follows: repetition time: 1,100 ms; echo time: 15 ms; field of view: 22 cm; matrix: 320 × 320; layer thickness: 0.6 mm; number of excitations: 1; scanning direction: coronal position; excitation mode: selective; voxel size: 0.6 mm × 0.6 mm × 0.6 mm; locs per slab: 90; scanning time: 5 min and 35s. During the scan, a gadolinium contrast agent (Magnevist) was used as an enhanced contrast medium.

The 3D-CUBE-T1 enhanced sequence data were imported into the ADW 4.7 and utilized workstation (General Electric Medical System, WI, USA) to reconstruct images with a slice thickness of 1.0 mm and a spacing between images of 1.0 mm. The cross-sectional images of carotid arteries were obtained by extending the bifurcation sites of ECA and ICA to the proximal and distal ends by 2.0 cm, respectively, perpendicular to the lumen axis of CCA, ECA, ICA, and Sinus. In addition, curved projection reformation along the lumen above the axis was performed to obtain a longitudinal view of the carotid artery.

### Image analysis

Two trained radiologists (with more than five years of clinical experience in MR imaging of carotid arteries) independently evaluated all collected HR-MRI images without accessing clinical information. We assessed the bilateral carotid arteries independently, and a complete carotid artery image should include a segment of CCA, ICA, ECA, and an intact Sinus for accurate measurement of the vessel wall and lumen. The quality of HR-MRI images was graded as excellent [high signal-to-noise ratio (SNR), no artifacts, complete suppression of intravascular blood flow signal, precise arterial wall contour, and a distinct boundary with the lumen], good (high SNR, with minimal motion artifacts, incomplete suppression of intravascular blood flow signal, precise arterial wall contour, and a distinct boundary with the lumen), marginal (low SNR, with motion artifacts, having arterial wall contour but indistinguishable wall structure), and poor (low SNR, with significant motion artifacts, unclear display of arterial blood vessels). Images graded as marginal and poor were excluded from this study.

Image analysis was performed using VesselMASS V2019-EXP software (Leiden University Medical Center, the Netherlands). After the eligible reconstructed images were imported into the software, the contours of the outer and inner vessel walls of the carotid artery in the image were manually delineated, followed by correction using the software. The morphometric parameters of CCA, ICA, ECA, and Sinus were measured separately in each layer. The total vessel area (TVA), lumen area (LA), wall thickness (WT), wall area (WA, WA = TVA − LA), normalized wall index (NWI, NWI = WA/TVA × 100%), and lumen area ratio of ICA/CCA (LA_ICA_/LA_CCA_-ratio) were measured and calculated. The highest value of WT measured in all slices (WTmax) was selected. The bifurcation angle (BIFA) was defined as the angle between the inner wall of the ECA and ICA measured on a longitudinal view image obtained by curved projection reformation. In addition, we described concentric and eccentric enhancement of the vessel walls to assess vasculitis and atherosclerotic plaques (30, 31). Enhancement was recorded as concentric if it was uniform and involved the entire circumference of the vessel wall and as eccentric if it was nonuniform, mainly on one side of the vessel wall and not involving the entire circumference (32).

### Null hypotheses

Our null hypotheses considered that patients in the thrombosis group had carotid morphometric parameters similar to those in the non-thrombosis group. A power calculation based on an article (18) and our pre-experiment results assessing the thickness of IMT (Doppler ultrasound) or vessel wall (MRI) of the Sinus in patients in the thrombosis group and non-thrombosis group revealed that a minimum of 29 to 32 arteries were required in each group for an *α* at 0.05 and a power of 80%.

### Statistical analysis

Statistical analyses were conducted using SPSS 19.0 software (IBM, USA). Data were presented as mean ± standard deviation for continuous variables and percentages for categorical variables. Independent Student's *t*-test was used to compare continuous data between two groups, and one-way analysis of variance was employed for comparisons involving more than two groups. Bonferroni correction was applied to adjust for multiple comparisons. The *χ*^2^ and Fisher's exact tests were used for univariable comparisons between categorical variables. Binary logistic regression analysis was performed to identify independent risk factors for thrombotic events, and the odds ratio (OR) and corresponding 95% confidence interval (CI) of each factor were calculated. The factors with significant differences in descriptive statistics between the thrombosis and non-thrombosis groups were analyzed using univariable binary logistic regression analysis, and the factors verified by univariable regression analysis were entered into the multivariable regression analysis. Significant independent risk factors were selected and plotted on a receiver operator characteristic (ROC) curve using Prism 9 software (GraphPad Software, USA), and the area under the curve (AUC), along with the cut-off value, was calculated. All results were presented as 2-tailed values, and a *P*-value of <0.05 was considered statistically significant.

## Results

### Study population

After applying the inclusion and exclusion criteria, 52 patients with SLE/aPLs were enrolled in this study, among whom 29 met the Revised Sapporo criteria of APS and 23 had positive aPL only. Of the 29 patients with APS, 24 met the thrombotic criteria only, 1 met the obstetric criteria only, and 4 met both the thrombotic and obstetric criteria. Among the 52 patients with SLE/aPLs, 28 patients with at least one well-documented thrombotic event or ongoing thrombosis were defined as the thrombosis group, as well as the other 24 patients without a history of thrombosis and thrombosis excluded by ancillary examinations were defined as the non-thrombosis group. Additionally, 20 healthy participants without any history of autoimmune disease and thrombosis were classified into the control group. The main demographic and clinical characteristics of the study population are shown in Table 1. There was no statistically significant difference in baseline characteristics between the thrombosis, non-thrombosis, and control groups (*P* > 0.05). The thrombosis group had a higher rate of antithrombotic therapy than the non-thrombosis group (Total: 75.0% vs. 45.8%, *P* = 0.031; Warfarin only: 39.3% vs. 0.0%, *P* = 0.001). Additionally, the thrombosis group had a higher number of positive aPLs (single positive: double positive: triple positive, 28.6%; 42.9%; 28.6% vs. 62.5%; 20.8%; 16.7%, *P* = 0.048) and LAC positive rate (64.3% vs. 25.0%, *P* = 0.005) compared with the non-thrombosis group. The APS “non-criteria” manifestations of the thrombosis and non-thrombosis groups are shown in Supplementary Table S1, and no significant difference was shown between these two groups (*P* > 0.05).

**Table 1:** Demographic and clinical data of patients with SLE/aPLs and healthy control.

	Thrombosis	Non-thrombosis	Control	*P*-value
	(*n* = 28)	(*n* = 24)	(*n* = 20)	
Demographic status				
Gender (female), *n* (%)	21 (75.0)	22 (91.7)	14 (70.0)	0.166
Age (years)	41.54 ± 11.37 (21–61)	35.79 ± 11.51 (20–60)	40.60 ± 13.71 (20–61)	0.209
Diabetes mellitus, *n* (%)	1 (3.6)	0 (0)	2 (10.0)	0.191
Hypertension, *n* (%)	9 (32.1)	4 (16.7)	3 (15.0)	0.269
Hyperlipidemia, *n* (%)	12 (42.9)	7 (29.2)	3 (15.0)	0.117
Smoking habit, *n* (%)	4 (14.3)	2 (8.3)	4 (20.0)	0.529
Obesity, *n* (%)	5 (17.9)	1 (4.2)	3 (15.0)	0.252
BMI (kg/m^2^)	25.85 ± 3.78	23.30 ± 4.03	24.85 ± 4.56	0.088
Course of disease (months)	92.25 ± 68.64 (12–249)	88.33 ± 87.22 (12–393)	–	0.857
Laboratory examination and disease activity				
CRP (mg/L)	2.05 ± 3.20	5.07 ± 7.12	–	0.062
ESR (>20 mm/h), *n* (%)	14 (50.0)	10 (41.7)	–	0.548
C3 (g/L)	0.95 ± 0.23	0.96 ± 0.19	–	0.855
C4 (g/L)	0.19 ± 0.09	0.18 ± 0.08	–	0.734
Anti-dsDNA (IU/ml)	105.86 ± 166.26	121.92 ± 187.86	–	0.745
SLEDAI-2K	2.43 ± 3.77	2.21 ± 4.29	–	0.845
APLs				
aCL positive, *n* (%)	16 (57.1)	15 (62.5)	–	0.695
High titer of aCL, *n* (%)	6 (21.4)	2 (8.3)	–	0.262
aβ2GPI positive, *n* (%)	22 (78.6)	16 (66.7)	–	0.335
LAC positive, *n* (%)	18 (64.3)	6 (25.0)	–	0.005
Number of positive aPLs, *n* (%)				
Single-positive	8 (28.6)	15 (62.5)	–	0.048
Double-positive	12 (42.9)	5 (20.8)	–	
Triple-positive	8 (28.6)	4 (16.7)	–	
Treatment				
Antithrombotic treatment, *n* (%)	21 (75.0)	11 (45.8)	–	0.031
Aspirin only, *n* (%)	9 (32.1)	9 (37.5)	–	0.686
Warfarin only, *n* (%)	11 (39.3)	0 (0)	–	0.001
Combination of Aspirin and Warfarin, *n* (%)	1 (3.6)	1 (4.2)	–	1.000
Statin, *n* (%)	7 (25.0)	1 (4.2)	–	0.091
Corticosteroids, *n* (%)	24 (85.7)	23 (95.8)	–	0.446
Hydroxychloroquine, *n* (%)	24 (85.7)	21 (87.5)	–	1.000
Immunosuppressant, *n* (%)	12 (42.9)	12 (50.0)	–	0.606
Biological agents, *n* (%)	14 (50.0)	11 (45.8)	–	0.764

SLE/aPLs, systemic lupus erythematosus with antiphospholipid antibodies; BMI, body mass index; aCL, anticardiolipin antibody; aβ2GPI, anti-β2glycoprotein antibody; LAC, lupus anticoagulant; aPLs, antiphospholipid antibodies; CRP, C-reactive protein; ESR, erythrocyte sedimentation rate; C3, complement C3; C4, complement C4; anti-dsDNA, anti-double-stranded DNA antibody; SLEDAI-2K, systemic lupus erythematosus disease activity index-2000.

### HR-MRI image features

#### The carotid arteries of patients with SLE/aPLs exhibited unique morphometric characteristics

A total of 144 carotid arteries were measured. Within the SLE/aPLs group, more patients in the thrombosis group had eccentric enhancement than those in the non-thrombosis group (16/56 (28.6%) vs. 3/48 (6.3%), *P* = 0.003), and the presence of concentric enhancement in the two groups was low (2/56 (3.6%) vs. 0/48 (0%), *P* = 0.498). Quantitative measurements and calculations of TVA, LA, WA, WT, WTmax, and NWI of the CCA, ICA, ECA, and Sinus, including global WTmax, LA_ICA_/LA_CCA_-ratio and BIFA were shown in Table 2. The WA, WT, and WTmax of CCA, ICA, and Sinus, together with TVA_CCA_, NWI_Sinus_, and WTmax_Global,_ all showed significant differences between the three groups (*P* < 0.05). WT, WTmax, and NWI of ECA, as well as NWI_CCA_ and NWI_ICA_, showed statistically significant differences between the thrombosis group and the control group and between the non-thrombosis group and the control group (*P* < 0.05) but not significant between the thrombosis and non-thrombosis groups (*P* > 0.05). The TVA_ICA_, TVA_Sinus_, and BIFA only demonstrated significant differences between the thrombosis and control groups (*P* < 0.05).

**Table 2:** Comparison of morphometric characteristics between carotid arteries of patients with SLE/aPLs and healthy control.

	Thrombosis (*n* = 56)	Non-thrombosis (*n* = 48)	Control (*n* = 40)	*P*-value of thrombosis vs. non-thrombosis	*P*-value of thrombosis vs. control	*P*-value of non-thrombosis vs. control
CCA						
TVA, mm^2^	85.52 ± 22.98	73.73 ± 21.86	63.00 ± 14.70	0.013	<0.001	0.049
LA, mm^2^	38.86 ± 9.66	35.67 ± 10.43	34.98 ± 6.81	0.245	0.133	1.000
WA, mm^2^	45.93 ± 15.42	38.04 ± 13.01	28.03 ± 9.60	0.009	<0.001	0.002
WT, mm	1.68 ± 0.40	1.46 ± 0.30	1.10 ± 0.23	0.003	<0.001	<0.001
WTmax, mm	3.14 ± 1.19	2.46 ± 0.69	1.65 ± 0.30	<0.001	<0.001	<0.001
NWI, %	52.98 ± 6.98	51.13 ± 5.90	43.60 ± 6.52	0.447	<0.001	<0.001
ICA						
TVA, mm^2^	66.88 ± 19.10	59.54 ± 20.94	53.45 ± 12.50	0.128	0.002	0.362
LA, mm^2^	33.23 ± 12.05	31.25 ± 12.07	31.60 ± 5.96	1.000	1.000	1.000
WA, mm^2^	33.63 ± 10.74	28.27 ± 10.39	21.85 ± 8.33	0.022	<0.001	0.010
WT, mm	1.35 ± 0.34	1.19 ± 0.25	0.94 ± 0.22	0.012	<0.001	<0.001
WTmax, mm	2.41 ± 1.18	1.84 ± 0.75	1.36 ± 0.29	0.003	<0.001	0.035
NWI, %	50.48 ± 8.60	48.02 ± 6.67	39.78 ± 7.38	0.314	<0.001	<0.001
ECA						
TVA, mm^2^	31.82 ± 11.49	27.67 ± 10.78	27.20 ± 8.11	0.133	0.101	1.000
LA, mm^2^	14.86 ± 5.28	12.67 ± 5.36	14.30 ± 4.21	0.086	1.000	0.396
WA, mm^2^	16.96 ± 7.15	15.00 ± 6.11	12.85 ± 4.71	0.329	0.005	0.323
WT, mm	0.99 ± 0.25	0.96 ± 0.22	0.81 ± 0.20	1.000	0.001	0.010
WTmax, mm	1.48 ± 0.38	1.42 ± 0.31	1.22 ± 0.30	0.961	0.001	0.026
NWI, %	52.64 ± 7.73	53.98 ± 6.96	46.75 ± 6.82	1.000	<0.001	<0.001
Sinus						
TVA, mm^2^	109.36 ± 24.16	101.15 ± 28.12	89.88 ± 24.79	0.320	0.001	0.127
LA, mm^2^	53.14 ± 16.04	53.54 ± 18.64	53.48 ± 14.66	1.000	1.000	1.000
WA, mm^2^	56.38 ± 13.48	47.75 ± 13.23	36.40 ± 12.83	0.003	<0.001	<0.001
WT, mm	1.78 ± 0.36	1.53 ± 0.28	1.16 ± 0.23	<0.001	<0.001	<0.001
WTmax, mm	3.55 ± 1.34	2.70 ± 0.85	1.78 ± 0.37	<0.001	<0.001	<0.001
NWI, %	51.71 ± 7.45	47.56 ± 7.62	39.75 ± 6.62	0.013	<0.001	<0.001
Global						
LA_ICA_/LA_CCA_-ratio, %	86.39 ± 26.96	88.56 ± 25.37	93.35 ± 23.81	1.000	0.574	1.000
WTmax, mm	3.76 ± 1.37	3.00 ± 0.93	1.92 ± 0.29	0.001	<0.001	<0.001
BIFA, °	47.66 ± 15.56	41.35 ± 19.25	36.30 ± 15.06	0.173	0.004	0.483

SLE/aPLs, systemic lupus erythematosus with antiphospholipid antibodies; CCA, common carotid artery; ICA, internal carotid artery; ECA, external carotid artery; Sinus, carotid bulb; TVA, total vessel area; LA, lumen area; WA, wall area; WT, wall thickness; WTmax, the highest value of wall thickness measured in all slices; NWI, normalized wall index; LA_ICA_/LA_CCA_-ratio, lumen area ratio of internal carotid artery and common carotid artery; BIFA, bifurcation angle of internal carotid artery and external carotid artery.

#### A negative LAC better distinguishes carotid morphometric changes in patients with SLE/aPLs without thrombotic events

We further stratified patients according to the status (positive or negative) of LAC that differed between the thrombosis and non-thrombosis groups (Table 1) and performed statistical analysis (Table 3). The results showed that LAC-positive patients had higher WTmax_CCA_ than LAC-negative patients in the thrombosis group (3.35 ± 1.32 mm vs. 2.76 ± 0.79 mm, *P* = 0.041). LAC-positive patients showed higher TVA_CCA_, WA_CCA_, WT_CCA_, and WTmax_CCA_ than LAC-negative patients in the non-thrombosis group (*P* < 0.05). In contrast, in the LAC-positive group, patients with thrombotic events presented higher WT, WTmax, and NWI in the Sinus than those without thrombotic events (*P* < 0.05). Among LAC-negative patients, the TVA, WA, WT, and WTmax of CCA and ICA, as well as WT_Sinus_, were higher in patients combined with thrombotic events than patients without thrombotic events (*P* < 0.05). It can be concluded that a negative LAC (with or without positive aCL or aβ2GPI) contributed to illustrating lower increased TVA and thickened vessel walls of CCA and ICA in SLE/aPLs patients without thrombotic events.

**Table 3:** Comparison of morphometric characteristics between carotid arteries of patients with SLE/aPLs accompanying positive or negative LAC.

	LAC-positive (*n* = 48)			LAC-negative (*n* = 56)			*P*-value of thrombosis with LAC + vs. thrombosis with LAC–	*P*-value of non-thrombosis with LAC + vs. non-thrombosis with LAC–
	Thrombosis (*n* = 36)	Non- thrombosis (*n* = 12)	*P*- value	Thrombosis (*n* = 20)	Non- thrombosis (*n* = 36)	*P*- value		
CCA								
TVA, mm^2^	86.75 ± 25.01	87.25 ± 21.86	0.951	83.30 ± 19.21	69.22 ± 20.20	0.014	0.595	0.012
LA, mm^2^	38.89 ± 9.87	40.75 ± 9.39	0.570	38.80 ± 9.53	33.97 ± 10.33	0.091	0.974	0.050
WA, mm^2^	46.72 ± 17.39	46.50 ± 13.61	0.968	44.50 ± 11.28	35.22 ± 11.68	0.006	0.510	0.008
WT, mm	1.69 ± 0.44	1.65 ± 0.30	0.757	1.65 ± 0.30	1.40 ± 0.28	0.003	0.678	0.010
WTmax, mm	3.35 ± 1.32	2.83 ± 0.60	0.073	2.76 ± 0.79	2.34 ± 0.68	0.042	0.041	0.030
NWI, %	52.93 ± 7.99	52.67 ± 5.19	0.920	53.10 ± 4.83	50.61 ± 6.10	0.122	0.926	0.301
ICA								
TVA, mm^2^	64.19 ± 19.36	63.83 ± 17.61	0.955	71.70 ± 18.11	58.11 ± 21.98	0.022	0.161	0.418
LA, mm^2^	31.97 ± 12.49	32.42 ± 6.53	0.875	35.50 ± 11.16	30.86 ± 13.48	0.196	0.298	0.703
WA, mm^2^	32.22 ± 11.02	31.33 ± 13.35	0.819	36.15 ± 9.98	27.25 ± 9.21	0.001	0.192	0.243
WT, mm	1.32 ± 0.37	1.27 ± 0.38	0.665	1.41 ± 0.29	1.16 ± 0.19	<0.001	0.392	0.377
WTmax, mm	2.30 ± 1.11	2.19 ± 1.34	0.791	2.61 ± 1.30	1.72 ± 0.36	0.007	0.337	0.253
NWI, %	50.56 ± 9.81	47.75 ± 7.19	0.367	50.35 ± 6.10	48.11 ± 6.58	0.216	0.933	0.879
ECA								
TVA, mm^2^	31.83 ± 11.55	29.67 ± 12.89	0.587	31.80 ± 11.67	27.00 ± 10.10	0.113	0.992	0.464
LA, mm^2^	15.03 ± 5.29	13.83 ± 7.09	0.538	14.55 ± 5.37	12.28 ± 4.71	0.105	0.749	0.390
WA, mm^2^	16.81 ± 6.85	15.83 ± 6.87	0.672	17.25 ± 7.84	14.72 ± 5.91	0.179	0.826	0.591
WT, mm	0.98 ± 0.24	0.98 ± 0.24	0.962	1.00 ± 0.28	0.95 ± 0.22	0.512	0.842	0.724
WTmax, mm	1.48 ± 0.41	1.48 ± 0.40	0.993	1.50 ± 0.35	1.40 ± 0.28	0.241	0.839	0.448
NWI, %	52.22 ± 6.57	53.67 ± 8.64	0.546	53.40 ± 9.62	54.08 ± 6.45	0.778	0.629	0.860
Sinus								
TVA, mm^2^	111.06 ± 27.35	104.42 ± 21.02	0.447	106.30 ± 17.23	100.06 ± 30.30	0.401	0.485	0.647
LA, mm^2^	53.67 ± 18.08	55.58 ± 12.67	0.736	52.20 ± 11.88	52.86 ± 20.36	0.879	0.746	0.666
WA, mm^2^	57.67 ± 14.28	48.83 ± 10.93	0.057	54.05 ± 11.90	47.39 ± 14.03	0.079	0.341	0.747
WT, mm	1.82 ± 0.38	1.54 ± 0.24	0.020	1.69 ± 0.33	1.52 ± 0.29	0.046	0.199	0.801
WTmax, mm	3.81 ± 1.40	2.62 ± 0.63	<0.001	3.09 ± 1.12	2.73 ± 0.92	0.204	0.051	0.716
NWI, %	52.25 ± 7.80	46.50 ± 4.74	0.021	50.75 ± 6.87	47.92 ± 8.39	0.203	0.476	0.474
Global								
LA_ICA_/LA_CCA_-ratio, %	83.69 ± 30.83	81.17 ± 15.32	0.711	91.25 ± 17.73	91.03 ± 27.66	0.974	0.250	0.248
WTmax, mm	3.98 ± 1.40	3.25 ± 1.05	0.102	3.37 ± 1.24	2.91 ± 0.88	0.118	0.106	0.285
BIFA, °	46.64 ± 15.38	47.33 ± 17.44	0.896	49.50 ± 16.11	39.36 ± 19.65	0.054	0.515	0.218

SLE/aPLs, systemic lupus erythematosus with antiphospholipid antibodies; LAC, lupus anticoagulant; CCA, common carotid artery; ICA, internal carotid artery; ECA, external carotid artery; Sinus, carotid bulb; TVA, total vessel area; LA, lumen area; WA, wall area; WT, wall thickness; WTmax, the highest value of wall thickness measured in all slices; NWI, normalized wall index; LA_ICA_/LA_CCA_ -ratio, lumen area ratio of internal carotid artery and common carotid artery; BIFA, bifurcation angle of internal carotid artery and external carotid artery.

Since the number of positive aPLs varied between the thrombosis group and the non-thrombosis group (Table 1), we categorized all patients with SLE/aPLs into different aPLs-positive groups (single-positive, double-positive, and triple-positive) and performed statistical analysis (Supplementary Table S2). The results demonstrated statistical differences in specific indicators among the groups with varying numbers of positive aPLs. Compared to LAC, the discriminatory ability of the number of positive aPLs was limited. Additionally, we compared the carotid morphometric parameters of patients with high titer aCL with those with medium and low titer. The results showed that only the LA_ECA_ (16.69 ± 6.16 mm^2^ vs. 13.33 ± 5.12 mm^2^, *P* = 0.021), WTmax_Sinus_ (3.71 ± 1.13 mm vs. 3.06 ± 1.21 mm, *P* = 0.021), and BIFA (58.63° ± 19.31° vs. 42.23° ± 16.10°, *P* < 0.001) were with significant differences between patients with high aCL titer and with medium/low aCL titer (Supplementary Table S3).

#### Logistic regression analysis identified thickened WTmax_Sinus_ and WTmax_Global_ as independent risk factors for thrombotic events in patients with SLE/aPLs

We performed univariable and multivariable binary logistic regression analysis with thrombotic events as the outcome variable. The carotid morphometric parameters (Table 2) that were statistically different (*P* < 0.05) between the thrombosis group and the non-thrombosis group by univariable regression analysis (Table 4) were used as covariates to identify independent risk factors for thrombotic events in patients with SLE/aPLs. We adjusted age, gender, and BMI as confounding factors (model 1). The results showed that WTmax_ICA_ (OR = 5.207, 95% CI, 1.207−26.393, *P* = 0.046), WTmax_Sinus_ (OR = 5.654, 95% CI, 1.236−25.870, *P* = 0.026), and WTmax_Global_ (OR = 0.102, 95% CI, 0.018−0.589, *P* = 0.011) were independently associated with the concurrence of thrombotic events and incorporated into the final model (Table 4). Furthermore, we adjusted age, gender, BMI, course of disease, antithrombotic treatment, LAC, and number of positive aPLs (model 2). The results showed that WTmax_Sinus_ (OR = 10.054, 95% CI, 1.540–65.653, *P* = 0.016) and WTmax_Global_ (OR = 0.067, 95% CI, 0.009–0.520, *P* = 0.010) were independently associated with the concurrence of thrombotic events and incorporated into the final model (Table 4).

**Table 4:** Independent risk factors for SLE/aPLs combined with thrombotic events based on binary logistic regression analysis.

Variable	Univariable regression^a,b^		Multivariable model 1^a,c^		Multivariable model 2^a,d^	
	OR (95% CI*)*	*P*-value	OR (95% CI)	*P*-value	OR (95% CI)	*P*-value
CCA						
TVA	1.025 (1.005–1.045)	0.012	1.100 (1.000–1.209)	0.050	1.100 (0.985–1.228)	0.090
WA	1.042 (1.010–1.075)	0.010	0.851 (0.708–1.022)	0.084	0.816 (0.661–1.008)	0.059
WT	6.037 (1.748–20.846)	0.004	1.961 (0.065–59.286)	0.699	4.786 (0.135–170.141)	0.390
WTmax	2.236 (1.344–3.719)	0.002	3.049 (0.940–9.898)	0.063	2.444 (0.622–9.596)	0.200
ICA						
WA	1.053 (1.010–1.098)	0.016	0.947 (0.816–1.100)	0.476	1.014 (0.846–1.215)	0.879
WT	7.866 (1.570–39.399)	0.012	0.943 (0.004–215.321)	0.983	0.186 (0.001–177.168)	0.631
WTmax	2.117 (1.172–3.826)	0.013	5.207 (1.027–26.393)	0.046	5.320 (0.761–37.201)	0.092
Sinus						
WA	1.054 (1.018–1.091)	0.003	0.951 (0.872–1.038)	0.263	0.959 (0.868–1.060)	0.413
WT	15.693 (3.190–77.206)	0.001	63.529 (0.369–10,948.484)	0.114	33.627 (0.129–8,770.376)	0.216
WTmax	2.127 (1.361–3.323)	0.001	5.654 (1.236–25.870)	0.026	10.054 (1.540–65.653)	0.016
NWI	2,193.983 (6.996–688,038.557)	0.009	0.029 (0.001–25.575.669)	0.613	0.488 (0.001–2,288,641.255)	0.927
Global						
WTmax	1.816 (1.227–2.690)	0.003	0.102 (0.018–0.589)	0.011	0.067 (0.009–0.520)	0.010

SLE/aPLs, systemic lupus erythematosus with antiphospholipid antibodies; OR, odds ratio; CI, confidence interval; CCA, common carotid artery; ICA, internal carotid artery; Sinus, carotid bulb; TVA, total vessel area; WA, wall area; WT, wall thickness; WTmax, the highest value of wall thickness measured in all slices; NWI, normalized wall index; BMI, body mass index; LAC, lupus anticoagulant; aPLs, antiphospholipid antibodies.

^a^ The outcome variable of these regression models was the presence of thrombotic events.

^b^ The factors with significant differences (*P* < 0.05) in descriptive statistics between the thrombosis group and the non-thrombosis group in Table 2 were analyzed using univariable binary logistic regression.

^c^ Model 1 was adjusted for age, gender, and BMI.

^d^ Model 2 was adjusted for age, gender, BMI, course of disease, antithrombotic treatment, LAC, and number of positive aPLs.

**Figure 1:**
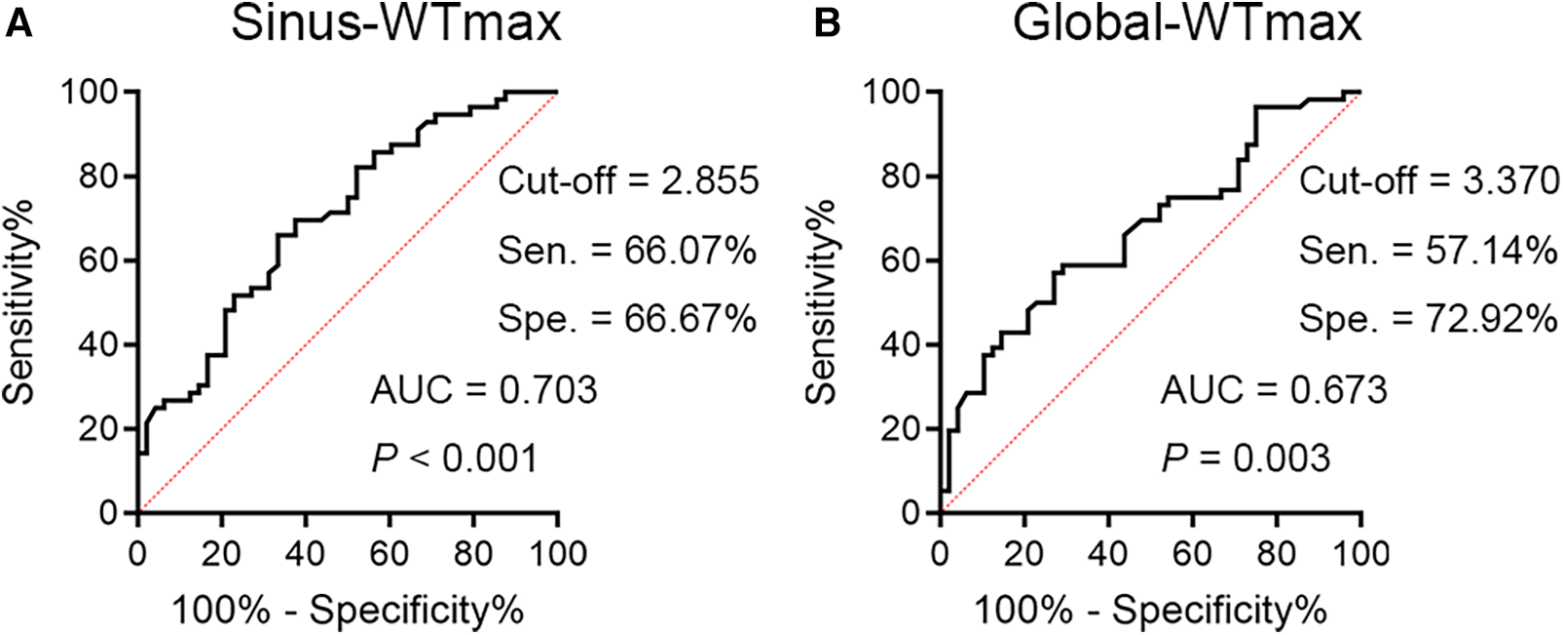
The ROC curves for WTmax_Sinus_ and WTmax_Global_ are shown. (**A**) The ROC curve for WTmax_Sinus_ has an AUC of 0.703, 95% CI 0.603–0.802, with a cut-off value of 2.855 mm (sensitivity = 66.07%, specificity = 66.67%). (**B**) The ROC curve for WTmax_Global_ has an AUC of 0.673, 95% CI 0.570–0.775, with a cut-off value of 3.370 mm (sensitivity = 57.14%, specificity = 72.92%). Sen, sensitivity; Spe, specificity.

Based on this, we further plotted ROC curves for WTmax_Sinus_ and WTmax_Global_. The AUC and cut-off values for each curve are shown in Figure 1. These data suggested that patients with SLE/aPLs who possessed a circumscribed thickened carotid vessel wall (>3.370 mm), particularly thickened at the Sinuses (>2.855 mm), should be careful with the occurrence of thrombotic events.

## Discussion

In this study, we explored the intricate relationship between morphometric parameters of carotid arteries and APS clinical phenotypes. Mechanistically, aPLs promote atherosclerosis and thrombosis in the body (33). The elevated occurrence rates of thrombotic events and atherosclerosis in SLE and APS patients have been extensively reported (6, 10, 12, 34, 35). However, these studies have been limited in reporting the incidence rates of thrombosis and atherosclerosis without exploring the correlation between the two. Research on the correlation between thrombosis and atherosclerosis is limited, and the conclusions are not yet clear (36–38). Therefore, we aim to establish a connection between thrombosis and atherosclerosis through imaging studies, providing a basis for vascular management and thrombosis prevention in the clinical diagnosis and treatment of SLE/aPLs patients.

Previous imaging studies of the carotid artery in APS patients primarily utilized Doppler ultrasound to observe and measure the vessel wall, with IMT as a key or sole data collection indicator (12, 13, 15, 18, 35). However, the experience and technique of the Doppler ultrasound operator significantly affect the measurement accuracy and standard consistency in different studies (39, 40). This study reconstructed HR-MRI images to present a clear, precise, and complete carotid artery morphology, ensuring accurate measurement of morphometric parameters. The clarity of the carotid vessel wall and lumen acquired by HR-MRI facilitates an overall understanding of characteristic morphometric changes, providing a solid foundation for accurately measuring morphometric parameters (20, 21, 41, 42). Consistent with previous reports based on carotid Doppler ultrasound and IMT measurement (12–15), HR-MRI observed accelerated atherosclerosis in SLE/aPLs patients compared to healthy participants.

**Figure 2:**
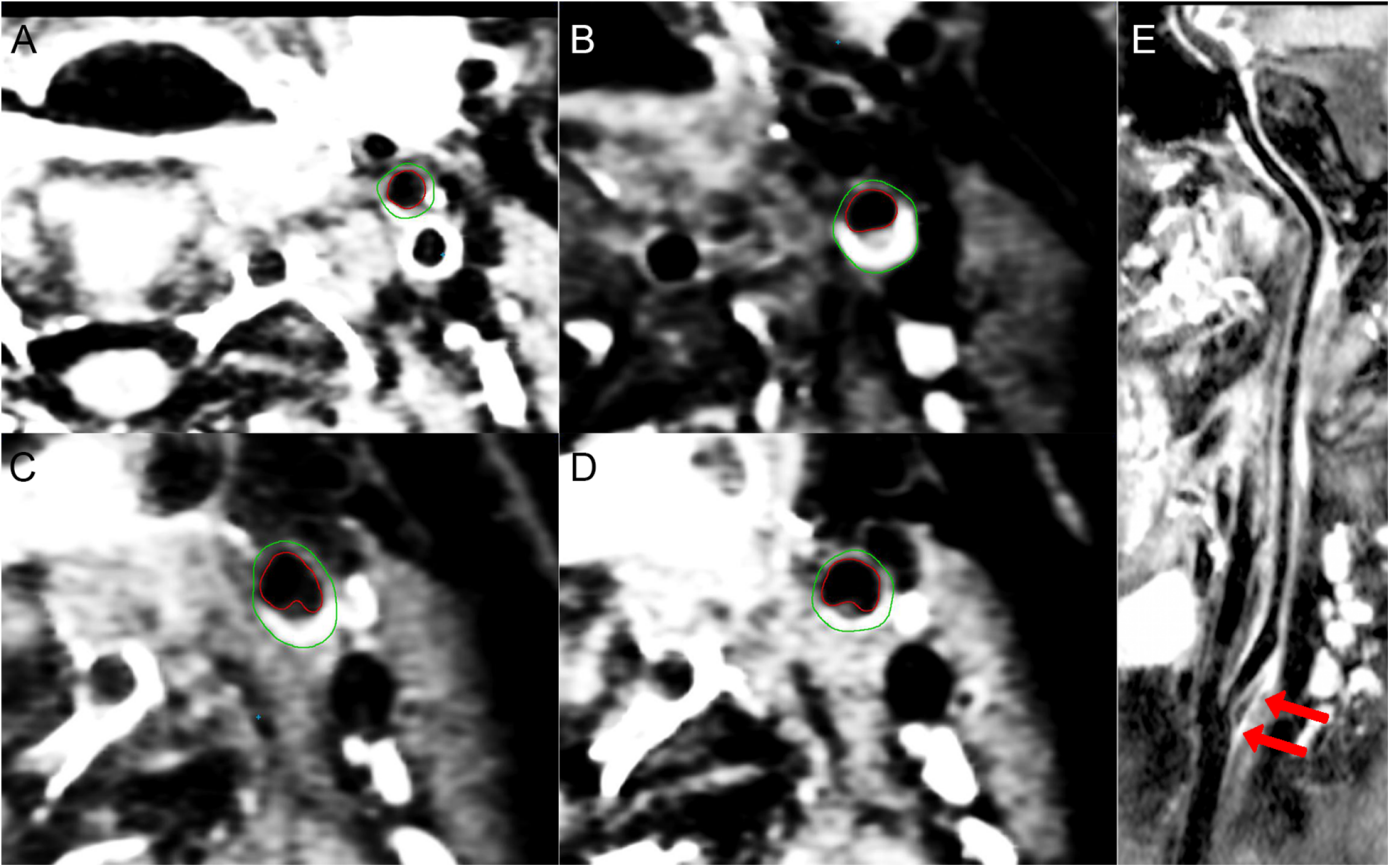
3D-CUBE-T1 weighted images of a 33-year-old woman presents with SLE/aPLs with positive aCL, aβ2GPI, and LAC combined with ischemic cerebral infarction in the left anterior circulation. (**A**–**D**) The contours of the outer (green) and inner (red) vessel walls of the left ECA (**A**), ICA (**B**), sinus (**C**), and CCA (**D**), respectively. Eccentric thickening and enhancement of the vessel wall can be seen at the carotid bifurcation (**C**) and the origin of the ICA (**B**). (**E**) longitudinal view image of curved projection reformation of the left carotid artery showing diffuse vessel wall thickening and atherosclerotic plaque formation at the origin of the left ICA (arrows). The BIFA of ICA-ECA is 38°.

The carotid artery is a susceptible site for atherosclerosis and serves as a crucial window for predicting and assessing the trends of atherosclerosis in the coronary and cerebral arteries (21, 43–46). In this study, we systematically measured the morphometric parameters of the carotid arteries in SLE/aPLs patients using HR-MRI. Our data suggested that the carotid arteries in patients with SLE/aPLs were primarily characterized by vessel wall thickening at CCA, ICA, ECA, and Sinus, and the patients with thrombotic events showed additional higher TVA of CCA and ICA, and BIFA of ICA-ECA without significant change in LA (Figure 2). The carotid arteries of those with thrombotic events exhibited significant vessel wall thickening in all segments except ECA compared to those without thrombotic events. Consistent with this, Yeo et al. also described diffuse proliferative vasculopathy and vessel wall thickening in the carotid arteries in APS patients (47). Overall, a specific correlation exists between thrombosis and atherosclerosis progression in patients with SLE/aPLs.

Studies by Ajeganova et al. (6), Tektonidou et al. (10), and Kravvariti et al. (12) have reported a higher prevalence of carotid atherosclerosis in SLE/aPLs patients. Our results also demonstrate thickening of the vessel walls in CCA, ICA, ECA, and Sinus in patients with SLE/aPLs compared to the healthy participants. Moreover, patients in the thrombosis group exhibited further thickening of the CCA, ICA, and Sinus vessel walls, elevated TVA_CCA_, and higher incidence of eccentricity enhancement (which generally means atherosclerotic plaque) than the non-thrombosis group. Consistent with this, Di Minno et al.'s study also observed a higher incidence of atherosclerotic plaques in patients with APS and thrombosis (13). Bettiol et al. reported that the thickness of CCA-IMT, Bulb-IMT, and the incidence of arterial atherosclerosis plaques in the APS thrombus group was significantly higher than in the APS obstetrics and control groups (18). Logistic regression analysis showed that WTmax_Sinus_ and WTmax_Global_ were independent risk factors for thrombotic events in patients with SLE/aPLs. Based on these results, we hypothesize that SLE/aPLs promotes thrombosis and atherosclerosis more substantially than traditional risk factors. The maximum thickness of the vessel wall of the artery is a crucial and intuitive indicator for evaluating atherosclerosis, reflecting the infiltration and destruction process of autoimmune inflammation on the vasculature. The thrombosis, which is also related to the inflammation process of SLE/aPLs, is thus linked to atherosclerosis. Particularly, we want to point out that patients with SLE commonly have small-vessel vasculitis (48, 49). In contrast, reports of large-vessel vasculitis are rare (50). So, we believe this may be why we only collected two cases of patients with carotid artery vasculitis (concentric enhancement). At present, researchers believe that SLE and APS promote carotid atherosclerosis, as mentioned above, rather than large-vessel vasculitis.

Furthermore, the results showed that both LAC-negative and non-thrombotic events distinguished relatively early atherosclerosis in the carotid arteries in patients with SLE/aPLs. The close association between LAC and thrombosis has been widely reported and accepted (51–53). However, limited studies are exploring the association between LAC and atherosclerosis. In a large multicentre population-wide study, LAC-positive women were at higher risk of stroke and myocardial infarction than LAC-negative women. Still, the authors did not specify whether atherosclerosis is the cause of stroke and myocardial infarction (54). The present study's results supplement and support a positive correlation between LAC and arterial lesions.

A positive number of aPLs (aCLs, aβ2GPI, and LAC) has been associated with APS symptoms, with a higher number of positive aPLs linked to increased IMT and atherosclerotic plaque incidence (12, 13, 18). The study by Yoo et al. reported that in patients with ischemic stroke, more than two positive aPLs predict persistent positive aPLs, which indicated the causes of ischemic stroke arising by APS (55), whereas, the present study observed limited intra- and inter-group morphometric parameter changes in the positive number of aPLs in the thrombosis and non-thrombosis groups. In addition, previous studies reported the correlation between aPL titer and atherosclerosis. A higher titer means a higher incidence of atherosclerosis (12, 13, 56). Reciprocally, our results showed little difference in carotid artery morphology between patients with high aCL titer and medium/low aCL titer. The possible reason may be that we only distinguished the titers of aCL [lacking convincing reference for the high titer threshold for detecting aβ2GPI using chemiluminescence (26)], and the sample size of patients with high aCL titer was relatively small in this study. Another point that needs to be explained is that this study focuses on whether there are differences in carotid morphometric parameters between SLE/aPLs patients with and without thrombotic events. However, there was no difference in aCL titer between the thrombosis and non-thrombosis groups, which may suggest a weak correlation between higher aCL titer and thrombotic events in SLE/aPLs patients. In other words, the difference in carotid morphometric parameters between the thrombosis and non-thrombosis groups may not be due to higher aCL titer. Alternatively, the reasons mentioned above are only for our data and analysis. Considering the limited sample size of this study, we believe that a multicentre study or meta-analysis may help identify additional indicators of carotid morphometric parameters associated with the number of positive aPLs and higher aCL/aPLs titers.

Apart from the limitations described in the previous section, the limitations of this study may also include sample size restrictions, as our data did not demonstrate any differences in LA between the various groups. Furthermore, as a cross-sectional study, this research was limited to the measurement and statistical observation of morphometric parameters of the carotid arteries. Whether the morphometric changes reflected by these parameters are correlated with the clinical symptoms and prognosis, and whether timely medical intervention is necessary still requires further support from prospective cohort studies.

## Conclusion

In summary, HR-MRI ensures the complete and accurate measurement of carotid morphometric parameters. The carotid arteries in patients with SLE/aPLs are mainly characterized by diffusely thickened vessel walls, and the patients with thrombotic events showed additional higher TVA of CCA and ICA, and BIFA of ICA-ECA without significant change in LA. The carotid arteries of SLE/aPLs patients with thrombotic events exhibited significant vessel wall thickening in all segments except ECA compared to those without thrombotic events. LAC-negative and non-thrombotic events distinguish relatively early atherosclerosis in the carotid arteries in patients with SLE/aPLs. Patients with SLE/aPLs that possess circumscribed thickened carotid vessel walls (>3.370 mm), particularly thickened at the Sinus (>2.855 mm), may require management strategies for the risk of thrombotic events.

## Data Availability

The raw data supporting the conclusions of this article will be made available by the authors, upon reasonable request.
