# Supplementary material for: Risk factors for thrombotic events in systemic lupus erythematosus patients with antiphospholipid antibodies: insights from morphometric measurements of carotid arteries
Source: Front Cardiovasc Med. 2024 Jun 26;11:1373097. doi: 10.3389/fcvm.2024.1373097 (PMC11233733; doi:10.3389/fcvm.2024.1373097)
Supplement: Supplementary file 1 [file Table1.docx]

Supplementary Material

**Table S1.** Comparison of APS “non-criteria” manifestation between thrombosis and non-thrombosis groups

|  | Thrombosis  (*n* = 28) | Non-thrombosis  (*n* = 24) | *P*-value |
| --- | --- | --- | --- |
| Obstetric manifestation | 7 | 3 | 0.431 |
| Thrombocytopenia | 19 | 18 | 0.571 |
| Cardiac microvascular disease | 1 | 1 | 1.000 |
| Livedo reticularis | 7 | 10 | 0.202 |
| Valvular heart disease | 2 | 1 | 1.000 |
| Amaurosis fugax | 1 | 0 | 1.000 |
| Hemolytic anemia | 1 | 3 | 0.495 |
| Migraine | 0 | 1 | 0.462 |
| Pulmonary hypertension | 1 | 1 | 1.000 |
| Seizures | 4 | 1 | 0.446 |
| Coombs' test positivity | 2 | 5 | 0.301 |
| Ischemic necrosis of bone | 3 | 1 | 0.718 |
| Raynaud's phenomenon | 3 | 2 | 1.000 |

**Table S2.** Comparison of morphometric characteristics between carotid arteries of patients with SLE/aPLs and different numbers of positive aPLs

|  | Single-positive aPLs  (*n* = 46) | | | Double-positive aPLs  (*n* = 34) | | | Triple-positive aPLs  (*n* = 24) | | |
| --- | --- | --- | --- | --- | --- | --- | --- | --- | --- |
|  | Thrombosis  (*n* = 16) | Non-thrombosis  (*n* = 30) | *P*-value | Thrombosis  (*n* = 24) | Non-thrombosis  (*n* = 10) | *P*-value | Thrombosis  (*n* = 16) | Non-thrombosis  (*n* = 8) | *P*-value |
| CCA | | | | | | | | | |
| TVA, mm^2^ | 84.50±19.17 | 69.83±22.07 | 0.030 | 86.54±23.97 | 74.10±13.18 | 0.063 | 85.00±26.18 | 87.88±25.96 | 0.802 |
| LA, mm^2^ | 39.38±10.11 | 34.40±11.15 | 0.144 | 39.25±9.16 | 35.10±6.37 | 0.202 | 37.75±10.46 | 41.13±11.06 | 0.472 |
| WA, mm^2^ | 45.13±10.78 | 35.40±12.66 | 0.012 | 46.88±17.99 | 39.00±8.23 | 0.090 | 45.31±16.01 | 46.75±16.35 | 0.839 |
| WT, mm | 1.68±0.29 | 1.39±0.30 | 0.003 | 1.67±0.47 | 1.53±0.20 | 0.203 | 1.69±0.39 | 1.65±0.36 | 0.834 |
| WTmax, mm | 2.59±0.53 | 2.29±0.70 | 0.147 | 3.37±1.42 | 2.80±0.67 | 0.235 | 3.34±1.17 | 2.67±0.58 | 0.141 |
| NWI, % | 53.25±4.93 | 50.37±6.11 | 0.112 | 52.71±7.88 | 52.40±4.90 | 0.910 | 53.13±7.68 | 52.38±6.44 | 0.815 |
| ICA | | | | | | | | | |
| TVA, mm^2^ | 70.88±19.07 | 59.47±23.77 | 0.105 | 62.71±19.24 | 54.70±8.94 | 0.108 | 69.13±18.87 | 65.88±20.57 | 0.703 |
| LA, mm^2^ | 35.00±11.92 | 32.03±14.50 | 0.487 | 30.13±13.01 | 28.50±6.08 | 0.623 | 36.13±10.14 | 31.75±6.78 | 0.284 |
| WA, mm^2^ | 35.88±10.47 | 27.43±9.88 | 0.010 | 32.54±11.49 | 26.20±4.92 | 0.104 | 33.00±10.15 | 34.00±15.58 | 0.851 |
| WT, mm | 1.40±0.31 | 1.15±0.20 | 0.002 | 1.36±0.41 | 1.16±0.17 | 0.144 | 11.28±0.27 | 1.36±0.44 | 0.610 |
| WTmax^1^, mm | 2.48±1.20 | 1.73±0.38 | 0.027 | 2.43±1.31 | 1.65±0.22 | 0.009 | 2.31±0.99 | 2.48±1.58 | 0.748 |
| NWI, % | 50.63±6.53 | 47.53±6.96 | 0.150 | 52.42±11.01 | 48.00±5.31 | 0.126 | 47.44±5.21 | 49.88±7.47 | 0.360 |
| ECA | | | | | | | | | |
| TVA, mm^2^ | 31.19±12.90 | 26.63±9.53 | 0.180 | 32.67±6.84 | 26.50±10.62 | 0.051 | 31.19±15.60 | 33.00±14.83 | 0.788 |
| LA, mm^2^ | 13.94±5.48 | 12.07±4.54 | 0.222 | 15.17±3.90 | 12.20±4.96 | 0.071 | 15.31±6.89 | 15.50±8.05 | 0.953 |
| WA, mm^2^ | 17.25±8.61 | 14.57±5.52 | 0.271 | 17.50±4.58 | 14.30±6.43 | 0.110 | 15.88±8.91 | 17.50±7.89 | 0.667 |
| WT, mm | 1.00±0.29 | 0.95±0.21 | 0.464 | 1.02±0.21 | 0.94±0.24 | 0.371 | 0.93±0.28 | 1.02±0.25 | 0.429 |
| WTmax, mm | 1.45±0.36 | 1.39±0.27 | 0.577 | 1.59±0.36 | 1.42±0.33 | 0.202 | 1.37±0.43 | 1.51±0.42 | 0.446 |
| NWI, % | 54.19±9.25 | 54.40±6.33 | 0.927 | 53.33±7.82 | 53.30±8.31 | 0.991 | 50.06±5.42 | 53.25±8.26 | 0.267 |
| Sinus | | | | | | | | | |
| TVA, mm^2^ | 103.50±17.06 | 101.27±30.92 | 0.791 | 110.38±27.96 | 98.70±25.27 | 0.263 | 113.69±24.34 | 103.75±22.60 | 0.345 |
| LA, mm^2^ | 51.63±11.09 | 53.67±19.87 | 0.706 | 52.83±19.87 | 52.80±20.09 | 0.996 | 55.13±14.44 | 54.00±13.35 | 0.855 |
| WA, mm^2^ | 51.81±8.67 | 47.87±14.95 | 0.338 | 57.54±15.92 | 45.80±8.75 | 0.036 | 59.19±13.04 | 49.75±11.88 | 0.100 |
| WT, mm | 1.63±0.18 | 1.52±0.29 | 0.153 | 1.82±0.47 | 1.51±0.28 | 0.054 | 1.85±0.29 | 1.59±2.44 | 0.037 |
| WTmax^2^, mm | 2.87±0.91 | 2.72±0.89 | 0.591 | 3.63±1.56 | 2.63±0.93 | 0.068 | 4.11±1.10 | 2.72±0.71 | 0.004 |
| NWI, % | 50.13±0.46 | 47.60±8.03 | 0.183 | 52.54±9.83 | 47.30±8.78 | 0.154 | 52.06±5.52 | 47.75±4.83 | 0.074 |
| Global | | | | | | | | | |
| LA_ICA_/LA_CCA_-ratio^3^, % | 88.06±14.92 | 93.33±29.43 | 0.425 | 76.29±27.49 | 81.20±9.90 | 0.451 | 99.88±30.41 | 79.88±18.58 | 0.104 |
| WTmax, mm | 3.19±1.16 | 2.92±0.85 | 0.377 | 3.91±1.51 | 2.99±0.91 | 0.086 | 4.12±1.22 | 3.29±1.26 | 0.130 |
| BIFA, ° | 48.81±16.01 | 40.50±21.30 | 0.179 | 45.96±12.60 | 39.40±8.57 | 0.143 | 49.06±19.49 | 47.00±21.70 | 0.816 |

SLE/aPLs: systemic lupus erythematosus with antiphospholipid antibodies, aPLs: antiphospholipid antibodies, CCA: common carotid artery, ICA: internal carotid artery, ECA: external carotid artery, Sinus: carotid bulb, TVA: total vessel area, LA: lumen area, WA: wall area, WT: wall thickness, WTmax: the highest value of wall thickness measured in all slices, NWI: normalized wall index, LA_ICA_/LA_CCA_-ratio: lumen area ratio of internal carotid artery and common carotid artery, BIFA: bifurcation angle of internal carotid artery and external carotid artery.

1 *P* = 0.030 between single-positive aPLs and triple-positive aPLs, and *P* = 0.048 between double-positive aPLs and triple-positive aPLs within the non-thrombosis group, respectively.

2 *P* = 0.024 between single-positive aPLs and triple-positive aPLs within the thrombosis group.

3 *P* = 0.018 between double-positive aPLs and triple-positive aPLs within the thrombosis group.

**Table S3.** Comparison of morphometric characteristics between carotid arteries of SLE/aPLs patients with high and medium/low titer of aCL

|  | High titer of aCL (> 80 GPL/MPL)  (*n* = 16) | Medium/low titer of aCL (≤ 80 GPL/MPL)  (*n* = 88) | *P*-value |
| --- | --- | --- | --- |
| CCA | | | |
| TVA, mm^2^ | 89.56±19.39 | 78.35±23.43 | 0.074 |
| LA, mm^2^ | 41.00±8.43 | 36.73±10.28 | 0.120 |
| WA, mm^2^ | 48.56±12.88 | 41.15±14.93 | 0.065 |
| WT, mm | 1.69±0.31 | 1.56±0.38 | 0.178 |
| WTmax, mm | 3.28±0.87 | 2.74±1.06 | 0.057 |
| NWI, % | 53.75±5.24 | 51.83±6.73 | 0.282 |
| ICA | | | |
| TVA, mm^2^ | 66.19±18.37 | 63.00±20.59 | 0.564 |
| LA, mm^2^ | 33.13±8.48 | 32.17±12.61 | 0.772 |
| WA, mm^2^ | 33.00±10.45 | 30.82±10.96 | 0.463 |
| WT, mm | 1.32±0.27 | 1.27±0.32 | 0.596 |
| WTmax, mm | 2.48±1.13 | 2.08±1.02 | 0.157 |
| NWI, % | 49.19±3.76 | 49.38±8.37 | 0.886 |
| ECA | | | |
| TVA, mm^2^ | 34.00±11.87 | 29.16±11.11 | 0.116 |
| LA, mm^2^ | 16.69±6.16 | 13.33±5.12 | 0.021 |
| WA, mm^2^ | 17.31±6.95 | 15.83±6.70 | 0.420 |
| WT, mm | 0.98±0.26 | 0.97±0.24 | 0.940 |
| WTmax, mm | 1.54±0.39 | 1.44±0.34 | 0.265 |
| NWI, % | 50.81±8.43 | 53.70±7.14 | 0.150 |
| Sinus | | | |
| TVA, mm^2^ | 114.13±17.26 | 104.01±27.37 | 0.157 |
| LA, mm^2^ | 56.81±11.91 | 52.69±17.98 | 0.381 |
| WA, mm^2^ | 57.94±14.28 | 51.39±13.77 | 0.085 |
| WT, mm | 1.80±0.41 | 1.64±0.33 | 0.086 |
| WTmax, mm | 3.71±1.13 | 3.06±1.21 | 0.047 |
| NWI, % | 50.50±8.31 | 49.67±7.72 | 0.697 |
| Global | | | |
| LA_ICA_/LA_CCA_-ratio, % | 83.44±25.78 | 88.11±26.28 | 0.513 |
| WTmax, mm | 3.80±1.01 | 3.34±1.27 | 0.172 |
| BIFA, ° | 58.63±19.31 | 42.23±16.10 | < 0.001 |

SLE/aPLs: systemic lupus erythematosus with antiphospholipid antibodies, aCL: anticardiolipin antibody, GPL: IgG phospholipid, MPL: IgM phospholipid, CCA: common carotid artery, ICA: internal carotid artery, ECA: external carotid artery, Sinus: carotid bulb, TVA: total vessel area, LA: lumen area, WA: wall area, WT: wall thickness, WTmax: the highest value of wall thickness measured in all slices, NWI: normalized wall index, LA_ICA_/LA_CCA_-ratio: lumen area ratio of internal carotid artery and common carotid artery, BIFA: bifurcation angle of internal carotid artery and external carotid artery.
